# Supplementary material for: Rational Design of a Polyurethane Foam
Source: Polymers (Basel). 2022 Nov 24;14(23):5111. doi: 10.3390/polym14235111 (PMC9736621; doi:10.3390/polym14235111)
Supplement: Supplementary file 1 [file polymers-14-05111-s001.zip › polymers-2052745-supplementary.pdf]

Table S1: Polyurethane foam formulations for experiments 1-4.

		Amount /PPHP								
Exp	Sample	Voranol 1447	Voranol 3322	Water	Vorasurf 5906	Vorasurf 5959	Dabco T	Cloisite	SpecFlex NE 112	Total
1	F05_01	75	25	4	1.5	0	0	5	81.10	191.60
	F05_02	75	25	4	1.5	0	0.3	5	81.22	192.02
	F05_03	75	25	4	1.5	0	0.6	5	81.34	192.44
	F05_04	75	25	4	1.5	0	0.9	5	81.46	192.86
	F05_05	75	25	4	1.5	0	1.2	5	81.58	193.28
	F05_06	75	25	4	1.5	0	1.5	5	81.70	193.70
	F05_07	75	25	4	1.5	0	1.8	5	81.82	194.12
	F05_08	75	25	4	1.5	0	2.1	5	81.94	194.54
2	F05_09	75	25	4	0	0	0.8	5	80.71	190.51
	F05_10	75	25	4	0.25	0	0.8	5	80.83	190.88
	F05_11	75	25	4	0.5	0	0.8	5	80.95	191.25
	F05_12	75	25	4	0.75	0	0.8	5	81.06	191.61
	F05_13	75	25	4	1	0	0.8	5	81.18	191.98
	F05_14	75	25	4	2	0	0.8	5	81.65	193.45
	F05_15	75	25	4	4	0	0.8	5	82.60	196.40
3	F05_28	75	25	4	1	0.1	0.8	5	81.26	192.16
	F05_29	75	25	4	1	0.2	0.8	5	81.34	192.34
	F05_30	75	25	4	1	0.3	0.8	5	81.42	192.52
	F05_31	75	25	4	1	0.5	0.8	5	81.57	192.87
	F05_32	75	25	4	1	1	0.8	5	81.96	193.76
	F05_33	75	25	4	1	2	0.8	5	82.74	195.54
	F05_34	75	25	4	1	0	0.8	5	81.18	191.98
	F05_35	75	25	4	1	3	0.8	5	83.52	197.32
	F05_36	75	25	4	1	4	0.8	5	84.30	199.10
4	F05_37	75	25	4	0.8	1.5	0.875	5	82.29	194.46
	F05_38	75	25	4	1.7	1	0.75	5	82.27	194.72
	F05_39	75	25	4	2	1	0.5	5	82.31	194.81
	F05_40	75	25	4	1.7	3	0.5	5	83.73	197.93
	F05_41	75	25	4	2	1	1	5	82.51	195.51
	F05_42	75	25	4	0.8	3	1	5	83.51	197.31
	F05_43	75	25	4	2	3	0.625	5	83.92	198.55
	F05_44	75	25	4	0.8	3	0.5	5	83.31	196.61
	F05_45	75	25	4	0.8	2.5	0.625	5	82.97	195.89
	F05_46	75	25	4	2	3	1	5	84.07	199.07
	F05_47	75	25	4	1.7	2	1	5	83.15	196.85
	F05_48	75	25	4	1.1	1.5	0.590335	5	82.32	194.51
	F05_49	75	25	4	0.8	1	1	5	81.95	193.75
	F05_50	75	25	4	1.1	2.590057	0.875	5	83.28	196.85
	F05_51	75	25	4	0.8	1	0.5	5	81.75	193.05
	F05_52	75	25	4	2	2.5	0.5	5	83.48	197.48
